# Exploring Structural and Vascular Changes of the Optic Nerve Head After Trabeculectomy in Primary Open-Angle Glaucoma

**DOI:** 10.3390/vision9040097

**Published:** 2025-12-07

**Authors:** Francesco Cappellani, Niccolò Castellino, Marco Zeppieri, Fabiana D’Esposito, Alessandro Avitabile, Giovanni Rubegni, Ludovica Cannizzaro, Giuseppe Gagliano, Antonio Longo

**Affiliations:** 1 Department of Medicine and Surgery, University of Enna “Kore”, Piazza dell’Università, 94100 Enna, Italy; 2 Department of Ophthalmology, University of Catania, 95123 Catania, Italy; 3 Department of Ophthalmology, University Hospital of Udine, 33100 Udine, Italy; 4 Department of Medicine, Surgery and Health Sciences, University of Trieste, 34127 Trieste, Italy; 5 Faculty of Medicine, University of Catania, 95123 Catania, Italy; 6 Ophthalmology Unit, Department of Medicine, Surgery and Neurosciences, University of Siena, 51300 Siena, Italy

**Keywords:** glaucoma, optic nerve head, Bruch’s membrane opening, lamina cribrosa, optical coherence tomography, OCT angiography, vessel density, retinal microvasculature, trabeculectomy

## Abstract

Background: Trabeculectomy remains gold-standard surgical approach for intraocular pressure (IOP) control in glaucoma, yet its impact on optic nerve head (ONH) morphology and retinal microvasculature has not been fully clarified. This study aimed to investigate structural and vascular changes of the ONH and macula after trabeculectomy using spectral-domain optical coherence tomography (SD-OCT) and OCT angiography (OCTA). Methods: In this retrospective study, data from 22 patients with primary open-angle glaucoma who underwent uncomplicated trabeculectomy were reviewed. The fellow eye served as control. Structural parameters, including Bruch’s membrane opening (BMO), maximum cup depth (MCD), and cup area, were measured with SD-OCT. Vessel density (VD) of the optic disc, peripapillary retina, and macular superficial (SCP) and deep (DCP) capillary plexuses were analyzed with OCTA. Preoperative and two-month postoperative data were compared using paired statistical tests. Results: Mean IOP decreased from 23.1 ± 3.9 mmHg to 13.2 ± 3.2 mmHg (*p* < 0.001). Significant postoperative reductions were observed in BMO (−5 ± 6%, *p* = 0.004), MCD (−31 ± 8%, *p* < 0.001), and cup area (−44 ± 18%, *p* < 0.001). RNFL thickness and ONH vascular parameters remained stable. In contrast, DCP vessel density increased in the foveal (*p* = 0.002) and parafoveal (*p* = 0.023) regions, while SCP density showed no significant change. Conclusions: Trabeculectomy was associated with measurable reversal of optic disc cupping, indicating partial structural recovery of the ONH following IOP reduction. The selective improvement in deep retinal vessel density suggests a layer-specific microvascular response. These findings provide further insight into the interplay between mechanical and vascular mechanisms in glaucoma and may inform postoperative monitoring strategies.

## 1. Introduction

Glaucoma is a heterogeneous group of diseases which lead to the progressive loss of retinal ganglion cells (RGC), excavation of the optic nerve head (ONH) and thinning of the retinal nerve fiber layer (RNFL), culminating in visual field loss [1,2,3]. It represents one of the leading causes of irreversible blindness worldwide, affecting over 76 million people globally as of 2020, with projections suggesting an increase to 111.8 million by 2040 [4,5]. Early diagnosis is essential to slow disease progression, but glaucoma often remains asymptomatic in its initial stages, and significant visual field damage may already be present by the time of diagnosis [1].

Several well-recognized risk factors contribute to the development and the progression of glaucoma, including advancing age, genetic predisposition, ethnicity, and the most significant and only modifiable which is elevated intraocular pressure (IOP) [6,7]. In addition to elevated intraocular pressure, several age-related anatomical changes contribute to increased glaucoma susceptibility. Progressive enlargement and thickening of the crystalline lens can narrow the anterior chamber angle and increase outflow resistance, thereby promoting IOP elevation. Moreover, reduced central corneal thickness, another recognized risk factor, may reflect the structural vulnerability of ocular tissues and influence both true IOP and individual biomechanical susceptibility to glaucomatous damage [8,9]. Effective management typically requires lifelong monitoring and treatment to prevent further deterioration of vision. Cataract surgery, by removing the enlarged lens, leads to deepening of the anterior chamber, widening of the iridocorneal angle, and improved aqueous outflow. Several studies have shown that phacoemulsification can lower IOP, reduce biomechanical stress across the lamina cribrosa, and potentially improve ocular perfusion dynamics in both open-angle and angle-closure disease [10,11,12].

Although the exact pathophysiology varies across different subtypes of glaucoma, elevated IOP is the most significant modifiable risk factor for disease progression [4]. However, glaucoma can also occur in patients with normal IOP levels, such as in normal-tension glaucoma (NTG), suggesting additional contributing factors such as vascular dysregulation and mechanical susceptibility of ONH tissues [13,14].

Two predominant theories attempt to explain glaucoma’s pathogenesis: the biomechanical theory and vascular theory. The vascular theory emphasizes the role of reduced ocular perfusion pressure, leading to ischemic and hypoxic damage to the optic nerve head [15]. In contrast, biomechanical theory suggests that elevated IOP induces stress and strain on the optic nerve head, causing mechanical damage to the lamina cribrosa (LC) and impairing axoplasmic flow through abnormally narrow scleral fenestrations [16,17,18].

The lamina cribrosa is a multilayered structure of connective tissue within the ONH and it serves as a biomechanical barrier between the intraocular space and the retrobulbar region, providing both structural and vascular support to the retinal ganglion cell axons as they exit the eye. However, this critical structure is susceptible to deformation under elevated IOP [19,20,21]. In glaucomatous eyes, the lamina cribrosa undergoes posterior displacement, thinning, and remodeling, which exacerbate the mechanical strain on the axons traversing its pores. These deformations may impede axoplasmic transport and contributing to glaucomatous damage [22,23].

Recent advancements in imaging technologies, including spectral-domain optical coherence tomography (SD-OCT) and optical coherence tomography angiography (OCTA), have revolutionized the assessment of ONH and peripapillary structures. SD-OCT provides high-resolution imaging of ONH morphology, allowing precise measurements of parameters such as Bruch’s membrane opening (BMO), maximum cup depth (MCD), and cup area. OCTA, on the other hand, enables non-invasive evaluation of vascular parameters such as retinal vessel density. These imaging modalities may provide a comprehensive view of the structural and vascular changes that occur in response to changes in IOP. Studies using these advanced imaging modalities have provided detailed insights into these structural changes, demonstrating significant posterior displacement of the LC in response to increased IOP [24,25].

Trabeculectomy is a surgical intervention for glaucoma management, primarily aimed at achieving long-term intraocular pressure IOP reduction to prevent or minimize progressive optic nerve damage and subsequent visual field loss. This procedure involves the formation of a regulated outflow pathway from the anterior chamber to the subconjunctival space, permitting aqueous humor to bypass the trabecular meshwork and drain directly into a subconjunctival filtration bleb. To enhance surgical outcomes and reduce scarring, adjunctive agents, such as mitomycin C (MMC) or 5-fluorouracil (5-FU) are frequently applied to modulate wound healing [26]. While trabeculectomy is widely recognized as one of the most effective surgical interventions for achieving IOP reduction, its specific impact on ONH morphology and vascular parameters remains an active area of investigation.

This study aims to evaluate the structural and vascular adaptations of the ONH following trabeculectomy in patients with POAG. By analyzing changes in parameters such as BMO, MCD, and vessel density using SD-OCT and OCTA, this research seeks to elucidate the relationship between IOP reduction and ONH remodeling. Understanding these dynamics will contribute to better surgical planning, enhanced monitoring strategies, and a deeper understanding of the biomechanical and vascular aspects of glaucoma pathophysiology.

## 2. Materials and Methods

In this retrospective observational analysis, we assessed consecutive patients who had been diagnosed with primary open-angle glaucoma (POAG) who underwent trabeculectomy at the Eye Clinic of the University of Catania between January 2023 and July 2024. The study adhered to the principles of the Declaration of Helsinki, and informed consent was obtained from all participants.

Patients were eligible for inclusion if they had a diagnosis of primary open-angle or pseudo-exfoliative glaucoma, had undergone uncomplicated trabeculectomy in one eye, and had high-quality imaging data available both before surgery and at the two-month postoperative follow-up. Exclusion criteria comprised angle-closure, neovascular or uveitic glaucoma, combined phacotrabeculectomy procedures, axial length greater than 26 mm, media opacities that impaired image quality, and spectral-domain OCT scans with signal strength below 7 or with segmentation errors that could not be corrected manually. The fellow eye of each patient served as control. All patients had a confirmed diagnosis of primary open-angle glaucoma or pseudo-exfoliative glaucoma, and all possible causes of unilateral glaucomatous optic neuropathy were assessed and excluded.

Comprehensive ophthalmic evaluation was performed preoperatively and two months after trabeculectomy. IOP was measured using Goldmann applanation tonometer. Central corneal thickness (CCT) was assessed using an ultrasound pachymeter, while axial length was obtained with the Zeiss IOLMaster device (Carl Zeiss Meditec AG, Jena, Germany).

For each patient, clinical and imaging data obtained at baseline (within one month before surgery) and at the postoperative visit approximately two months after trabeculectomy were reviewed. Only eyes with complete and comparable datasets from both time points were included. All images were acquired using standardized protocols and device settings routinely applied in our clinic.

**Figure 1:**
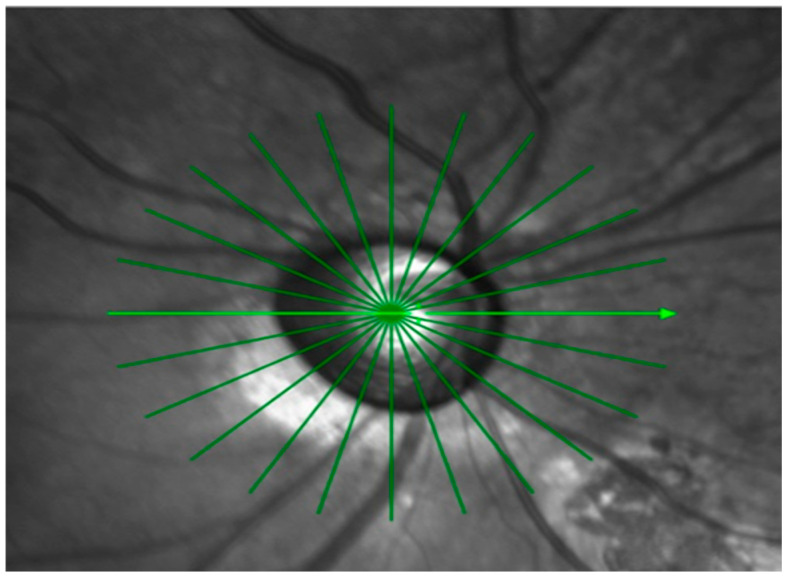
Optic nerve head radial scan centered on Bruch’s membrane opening (BMO) obtained using Spectralis SD-OCT. The 12-radial, 15° high-resolution scan pattern is shown.

OCT imaging of the optic nerve head ONH was performed with a Spectralis SD-OCT device (Heidelberg Engineering GmbH, Heidelberg, Germany). A radial scanning protocol was applied, consisting of twelve high-resolution 15-degree radial scans centered on BMO (Figure 1).

**Figure 2:**
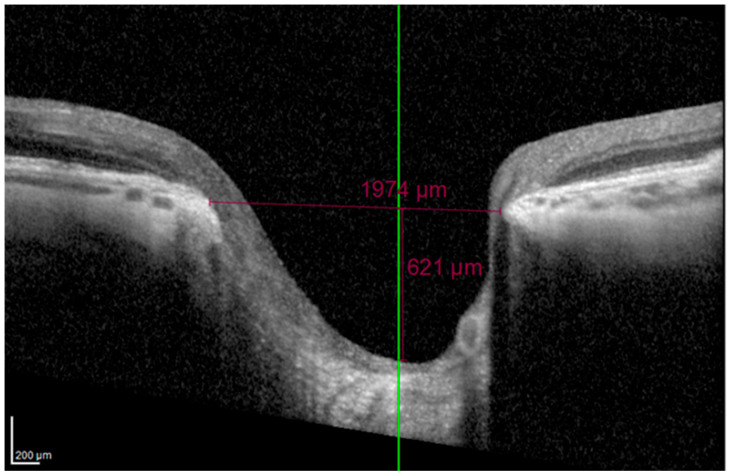
Measurement of Bruch’s membrane opening and maximum cup depth (MCD) on a horizontal SD-OCT scan.

On horizontal scans, the evaluators manually verified proper alignment of the scan on the ONH. The BMO was directly measured using the Heidelberg integrated software (Heidelberg Eye Explorer), and the MCD was determined along a perpendicular line drawn from the BMO plane to the deepest point of the cup (Figure 2).

**Figure 3:**
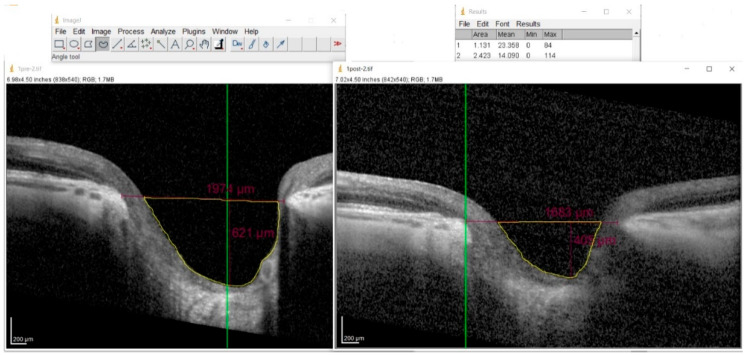
Determination of optic cup area using ImageJ software. The examiner manually outlines the cup margin on the SD-OCT image.

The cup area was computed using ImageJ software (NIH, Bethesda, MD, USA, version 1.54m). The operator manually outlined the borders of the cup, and the software automatically computed the enclosed area. The software’s Set Scale function was calibrated according to the manufacturer’s lateral and axial scaling factors provided by the Spectralis SD-OCT system (Figure 3).

OCTA was performed using the XR Avanti AngioVue system (Optovue, Fremont, CA, USA; version 2017.1.0.151, AngioVue Phase 7 Software with PAR). For optic disc angiography, a high-definition Angio Disc 4.5 × 4.5 mm scan was acquired. The program automatically outlined the disc margin with an elliptical fit and generated the corresponding mean vessel density (VD) inside the disc and the retinal circumpapillary (RCP) vessel density. For macular angiography, a high-definition Angio Retina 6 × 6 mm scan was performed. Two vascular layers were evaluated: the superficial capillary plexus (SCP), extending from 3 µm beneath the internal limiting membrane (ILM) to 15 µm below the inner plexiform layer (IPL), and the deep capillary plexus (DCP), located between 15 µm and 70 µm beneath the IPL.

Statistical analyses were conducted using SPSS software (version 26.0; IBM Corp., Armonk, NY, USA). Baseline parameters in study and fellow eyes were compared using the unpaired t-test. In study eyes, pre- and postoperative values were compared using the paired t-test. Correlations between changes in structural parameters and IOP reduction were assessed using Pearson’s correlation coefficient (r). Data are presented as mean ± standard deviation (SD), and statistical significance was defined as a *p*-value < 0.05.

## 3. Results

A total of 22 patients (9 men and 13 women; mean age 60 ± 6 years) who underwent trabeculectomy were included in the study. Mean follow-up period was 4 ± 1 months.

Baseline ocular parameters for study and fellow eyes are summarized in Table 1.

Following trabeculectomy, mean IOP decreased significantly from 23.1 ± 3.9 mmHg at baseline to 13.2 ± 3.2 mmHg at follow-up (*p* < 0.001). Significant reductions were also detected in BMO, MCD, and cup area (Table 2). The mean percentage decreases were −5 ± 6% for BMO, −31 ± 8% for MCD, and −44 ± 18% for cup area.

No significant postoperative changes were observed in RNFL thickness or in optic disc vessel density (inside disc and peripapillary) as measured by OCT angiography (Table 3).

Macular OCTA revealed no significant changes in SCP vessel density after trabeculectomy, although values remained consistently lower in study eyes than in fellow eyes. In contrast, DCP) vessel density increased significantly in the foveal and parafoveal regions following trabeculectomy, yet remained lower than in fellow eyes (Table 4).

## 4. Discussion

The objective of this investigation was to evaluate the morphological and vascular changes of the optic nerve and retina following trabeculectomy. Our findings revealed a significant reduction in Bruch’s membrane opening, maximal cup depth, and cup area after intraocular pressure reduction, while RNFL thickness and superficial capillary plexus vessel density remained unchanged. These results align with previous findings in the literature. Gietzelt et al. (2018) [27] found significant increases in BMO minimum rim width and area after trabeculectomy, both strongly correlated with IOP decrease, while RNFL thickness and visual field parameters remained stable. Lee et al. (2014) [28] investigated short-term changes in LC morphology following trabeculectomy employing enhanced-depth optical coherence tomography. Their findings revealed a significant anterior displacement of the LC after IOP reduction, with more pronounced changes observed in patients with greater preoperative LC depth and larger IOP reductions. Further studies have evaluated other ONH parameters. Park & Cha (2021) [29] reported that an increase in Bruch’s membrane opening minimum rim width was associated with younger age and greater IOP reduction. Ma et al. (2019) [30] investigated the deformation of ONH and of the peripapillary tissue (PPT) in reaction to acute IOP elevation. Using high-frequency ultrasonography, they imaged 14 human donor globes during inflation testing, where IOP was increased from 5 to 30 mmHg, and applied an algorithm to calculate tissue displacements. Their findings revealed that the ONH displaced more posteriorly than the PPT following an acute IOP increase. Although enlargement of the scleral canal was limited, it showed a consistent association with backward movement of the ONH at all tested IOP levels. Additionally, they observed a difference in how the optic nerve head and peripapillary tissue displaced, with more pronounced compressive forces observed in the anterior portions of the ONH and PPT, while shear forces were comparatively higher concentrated in the ONH periphery. Their later study (2020) [31] also showed that thinner peripapillary tissue is associated with greater posterior displacement. Sánchez et al. (2020) [32] also observed a reduction in cup depth and anterior LC surface depth after trabeculectomy, without significant change in RNFL thickness or BMO dimensions. Recent data from Shang et al. (2024) [33] further support this concept. They demonstrated a sustained reduction in the lamina cribrosa curvature index (LCCI) for more than three years after trabeculectomy, with younger age, lower postoperative IOP, and higher baseline LCCI identified as predictors of greater long-term change. These findings suggest that the ONH retains the ability to partially recover from mechanical deformation once stress from elevated IOP is relieved.

In our study, despite the morphological changes, RNFL thickness and optic disc vascular parameters, such as inside disc vessel density and peripapillary vessel density, did not show significant alterations post-trabeculectomy. These findings are consistent with those of Gietzelt et al. (2018) [27], who also observed structural changes in the ONH after IOP reduction without corresponding changes in RNFL thickness. Interestingly, while deep retinal capillary density increased in specific regions (e.g., fovea and perifovea) following trabeculectomy, superficial capillary plexus vessel density remained unchanged. This observation aligns with the unchanged RNFL thickness, as the superficial capillary plexus is linked to ganglion cell axons. The lamina cribrosa remains a focal point of glaucoma research, as its deformation under increased IOP is believed to play a central role in glaucomatous optic neuropathy. Mechanical models have demonstrated differential deformation of LC pores, potentially explaining the sectorial pattern of nerve fiber damage observed in glaucoma. The compression of laminar sheets and distortion of laminar pores reduce axoplasmic flow and disrupt capillary blood supply, leading to ischemic-hypoxic damage, growth factor deprivation, and altered cellular responses [16]. Beyond these mechanical insights, ongoing research is exploring cellular and molecular responses to IOP-induced LC deformation, offering potential avenues for novel strategies.

This study has several limitations. The primary one is the relatively small sample size, which may limit the generalizability of the findings. Second, the follow-up period was short, and long-term structural and vascular changes after trabeculectomy may differ from those observed at two months. Moreover, its retrospective design inherently limits control over potential confounding variables and precludes causal inference. Furthermore, visual field assessments were not included in the present analysis to determine whether the observed structural changes corresponded to functional improvement; therefore, future studies should incorporate serial visual field testing to address this important aspect. Finally, only eyes with uncomplicated trabeculectomy were included, and results may not apply to eyes undergoing combined or repeat surgical procedures.

## 5. Conclusions

Trabeculectomy induced significant structural remodeling of the optic nerve head, characterized by reductions in Bruch’s membrane opening, maximum cup depth, and cup area following intraocular pressure reduction. These morphological adaptations reflect the mechanical reversibility of lamina cribrosa deformation once the stress of elevated IOP is relieved. Despite these structural changes, retinal nerve fiber layer thickness and superficial vascular parameters of the optic nerve head and macula remained stable after surgery. The observed increase in deep capillary plexus vessel density suggests a selective microvascular response to pressure reduction, potentially reflecting improved perfusion in deeper retinal layers.

Understanding the biomechanical and vascular mechanisms underlying these changes is critical for improving surgical outcomes, refining postoperative management strategies, and advancing our knowledge of glaucoma pathophysiology. Further longitudinal studies with larger cohorts and extended follow-up are needed to confirm these observations and explore their functional implications.

## Figures and Tables

**Table 1:** Baseline ocular parameters in study and fellow eyes.

Parameter	Unit	Study Eyes	Fellow Eyes
Axial length	mm	24.7 ± 0.8	24.4 ± 0.9
Visual field MD	dB	−12.6 ± 2.8	−7.5 ± 3.9
CCT	µm	531 ± 13	543 ± 12

**Table 2:** Structural parameters in study eyes before and after trabeculectomy.

Parameter	Unit	Preoperative	Final	% Change	*p*-Value
IOP	mmHg	23.1 ± 3.9	13.2 ± 3.2	41 ± 18	<0.001
BMO	µm	1830 ± 109	1733 ± 58	−5 ± 6	0.004
MCD	µm	477 ± 110	332 ± 86	−31 ± 8	<0.001
Cup area	mm^2^	0.079 ± 0.022	0.043 ± 0.013	−44 ± 18	<0.001

**Table 3:** OCT and OCTA optic nerve parameters.

Parameter	Study Eyes (Pre-op)	Study Eyes (Final)	*p* vs. Pre-op	Fellow Eyes
RNFL thickness (µm)	73.3 ± 6.2	73.6 ± 5.8	0.708	111 ± 6
Inside disc VD (%)	44.7 ± 6.4	46.5 ± 6.2	0.394	51.3 ± 1.2
Peripapillary VD (%)	37.1 ± 5.2	39.2 ± 8.2	0.425	51.1 ± 2.0

**Table 4:** Macular vessel density measurements in the superficial and deep capillary plexuses.

Region	Parameter	Study Eyes (Pre-op)	Study Eyes (Final)	*p* vs. Pre-op	Fellow Eyes
Fovea	SCP VD (%)	6.6 ± 2.2	6.7 ± 1.4	0.865	10.2 ± 2.1
Parafovea	SCP VD (%)	36.9 ± 1.9	38.3 ± 3.1	0.347	52.9 ± 1.9
Perifovea	SCP VD (%)	34.5 ± 2.1	35.1 ± 2.3	0.729	50.4 ± 1.7
Fovea	DCP VD (%)	13.6 ± 3.9	20.9 ± 1.7	0.002	25.3 ± 2.7
Parafovea	DCP VD (%)	42.5 ± 3.7	46.2 ± 4.3	0.023	58.2 ± 2.7
Perifovea	DCP VD (%)	35.3 ± 1.1	41.1 ± 6.7	0.089	54.1 ± 3.3

## Data Availability

The data presented in this study are available on request from the corresponding author. The data are not publicly available due to privacy or ethical restrictions.

## Abbreviations

The following abbreviations are used in this manuscript:

IOP	Intraocular Pressure
ONH	Optic Nerve Head
SD-OCT	Spectral-Domain Optical Coherence Tomography
OCT	Optical Coherence Tomography
OCTA	Optical Coherence Tomography Angiography
BMO	Bruch’s Membrane Opening
MCD	Maximum Cup Depth
VD	Vessel Density
SCP	Superficial Capillary Plexus
DCP	Deep Capillary Plexus
RNFL	Retinal Nerve Fiber Layer
RGC	Retinal Ganglion Cell
NTG	Normal-Tension Glaucoma
LC	Lamina Cribrosa
ILM	Internal Limiting Membrane
IPL	Inner Plexiform Layer
CCT	Central Corneal Thickness
POAG	Primary Open-Angle Glaucoma
RCP	Retinal Circumpapillary
MD	Mean Deviation
MMC	Mitomycin C
5-FU	5-Fluorouracil
PPT	Peripapillary Tissue
LCCI	Lamina Cribrosa Curvature Index
